# Detecting activity locations from raw GPS data: a novel kernel-based algorithm

**DOI:** 10.1186/1476-072X-12-14

**Published:** 2013-03-16

**Authors:** Benoit Thierry, Basile Chaix, Yan Kestens

**Affiliations:** 1 Montreal University Hospital Research Center (CRCHUM), Pavillon Masson - 218 3850, St-Urbain, Montreal, H2W 1T7, Canada; 2 Inserm, Paris, U707, France; 3 Université Pierre et Marie Curie-Paris6, Paris, UMR-S 707, France; 4 Department of Social and Preventive Medicine, Université de Montréal, Montreal, Canada

**Keywords:** Global Positioning System (GPS), Activity location detection, Kernel-based algorithm, Neighbourhood effects, MHealth, Activity space

## Abstract

**Background:**

Health studies and mHealth applications are increasingly resorting to tracking technologies such as Global Positioning Systems (GPS) to study the relation between mobility, exposures, and health. GPS tracking generates large sets of geographic data that need to be transformed to be useful for health research. This paper proposes a method to test the performance of activity place detection algorithms, and compares the performance of a novel kernel-based algorithm with a more traditional time-distance cluster detection method.

**Methods:**

A set of 750 artificial GPS tracks containing three stops each were generated, with various levels of noise.. A total of 9,000 tracks were processed to measure the algorithms’ capacity to detect stop locations and estimate stop durations, with varying GPS noise and algorithm parameters.

**Results:**

The proposed kernel-based algorithm outperformed the traditional algorithm on most criteria associated to activity place detection, and offered a stronger resilience to GPS noise, managing to detect up to 92.3% of actual stops, and estimating stop duration within 5% error margins at all tested noise levels.

**Conclusions:**

Capacity to detect activity locations is an important feature in a context of increasing use of GPS devices in health and place research. While further testing with real-life tracks is recommended, testing algorithms’ performance with artificial track sets for which characteristics are controlled is useful. The proposed novel algorithm outperformed the traditional algorithm under these conditions.

## Introduction

Studies on the influence of contextual effects on health are increasingly resorting to tracking technologies such as Global Positioning Systems (GPS) to monitor mobility, which opens possibilities to continuously evaluate exposures to environmental conditions. Yet, GPS tracking generates a huge amount of geographic data which is tricky to handle in its raw form, and requires extraction of activity locations, and, possibly trips between activity locations, to be useful for health and place research. Various activity location algorithms have been proposed, but few metrics have been proposed to evaluate their performance. This paper has two aims: evaluate the performance of a novel kernel-based activity location detection algorithm, in comparison to a more classical detection method based on distance and time tresholds [1-3], and expose a novel method to evaluate the performance of such algorithms, using artificially generated tracks with known characteristics.

Identifying activity places from GPS can be considered an exercise of cluster detection, candidate locations being those where a sufficient number of data points are non-randomly distributed and have accumulated [4-7]. The classical approach for point cluster detection looks at the temporal sequence of recorded locations and uses a set of decision rules based on distance and time to identify clusters. This class of algorithms iteratively tests observations to determine if they remain within a given roaming distance of previous ones. If duration of stay within the distance threshold - time between the first and the last observed points - exceeds a predefined stay duration, the cluster is retained and its centroid is used as an approximation of stay location [1]. Similarly, Agamennoni et al. [8]. apply a speed threshold criteria. Using truck tracks in an open pit mine, they identify activity locations through low speed segments. Speed is also used in Biljecki [9] to determine stop places and to segment GPS data into trips. Ashbrook et al. [10] use a two-step procedure where, first, GPS points are flagged as significant places if the time interval with the previous point is below a certain threshold and, then, based on a distance criteria, clustered into locations.

Choosing the appropriate distance threshold is a challenge, its ideal value being a priori unknown, with possible needs for adjustment when adding new data [11]. Some learning algorithms have been proposed to optimise parameter choice, for example based on hierarchical conditional random fields [12] or Dirichlet process mixture models [7]. Liao et al. [12] use hierarchical conditional random fields that consider the high-level context to simultaneously derive activities and significant places from a person’s GPS track. Numri [7] proposes a method based on Dirichlet process mixture models, which functions well with spatio-temporal variations, to learn the number of places to extract and estimate the model parameters. Kami et al. [13] present a probabilistic place extraction algorithm based on density information that aims at minimizing the negative impact of GPS noise on the quality of the extracted places.

**Figure 1:**
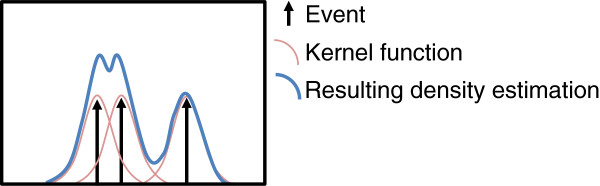
Kernel density estimation.

The hereby proposed algorithm differs from the traditional approach. It does not analyse datapoints sequentially. Rather, it uses GPS points to build a kernel density surface [14]. Kernel density estimation is a non-parametric method where a symmetrical kernel function is first superimposed over each event. The set of overlapping functions is then summed to create a continuous density surface (see Figure 1). Kernel densities are frequently used for point pattern analysis and hotspot exploration in a variety of domains, including criminology [15], spatial epidemiology [16-21] or ecology [22]. Local maxima are extracted from the kernel density surface and become activity location candidates for which time tables – periods of stay – are derived.

Both types of algorithms require the definition of a spatial and temporal parameter. The spatial parameter, or bandwidth value, corresponds to the roaming radius for the traditional method and the kernel bandwidth for the proposed algorithm. The temporal parameter defines the minimal duration of stay at a given location to qualify as a significant stop or activity location. An algorithm that would be less sensitive to bandwidth parameter value would be considered as more robust.

This paper assessed the performance of the proposed novel kernel-based activity location detection algorithm in comparison with the classical sequential distance threshold algorithm. Performance was assessed on a set of artificially generated GPS tracks. Use of artificial tracks permitted precise simulation of various GPS noise levels and activity stop durations, and consequently allowed performance testing under a large variety of conditions.

Analyses evaluated the algorithms’ capacity to detect (i) known activity locations and (ii) time spent at a given location, depending on (i) algorithm bandwidth value, (ii) GPS noise level, and (iii) actual stop duration. Our hypothesis was that the kernel-based algorithm would outperform the more classical cluster analysis. Particularly, we hypothesized that this novel method would be less susceptible to noise, due to the smoothing effect of kernel density estimations. For the same reason, we also thought the proposed algorithm would be less sensitive to bandwidth value, with high performance for a larger range of parameter values.

The proposed algorithm is currently used in active research projects and clinical interventions, including the Dyn@mo lifestyle intervention targeting children with cardiometabolic risk factors which makes use of GPS tracking to improve counselling [23] the RECORD-GPS study looking at mobility, exposures, and cardiovascular outcomes [24], and a project looking at the impact of a bicycle sharing program in Montreal [25].

### Why are temporal series of activity locations an important information for health and place research?

Studies of contextual effects on health have largely focused on place of residence, a clear limitation as most people spend a large portion of their time out of their residential environment [26,27]. Acquiring detailed information on people’s everyday activity locations and trips allows researchers to draw a more complete picture of true exposure to environmental hazards or experienced accessibility to opportunities [28-31]. Yet, why should we focus on extracting information on activity locations? The fact is that linking exposure and behaviour at all times, as would allow continuous GPS monitoring, does not tell us much about how structure opportunities influence behaviour and health, mainly because of selective daily mobility bias [32]. One of the ways to overcome such bias is to evaluate whether a behaviour of interest, observed at a given location and time – for example physical activity – is influenced by the exposure or structure of opportunities observed at the *previous or next activity location*, where accessibility to resources may have influenced use of resources and behaviour. This requires transforming GPS data into the sequence, the location and the nature of conducted activities. For example, if one wanted to know whether exposure to parks influenced park usage, the predictor of interest would be exposure to parks at all locations except where the behaviour of interest – park usage – is observed. More particularly, relevant exposure would be at the previous location, the subsequent location, or in the path between both locations. In short, in line with the principles of opportunity structures [33], relational approach [34], and space-time geography [35], a given activity or visit of an activity location is theoretically linked to the exposure or accessibility and the space-time budget at the previous or subsequent activity location.

Consequently, mobility tracking is useful for causality assessment in epidemiological studies as long as activity locations and the nature of activities – and not only raw mobility tracks – are identified. Transformation of GPS tracks into relevant spatial information has mostly been driven by the fields of transportation and mobile communication, with a focus on the automatic detection of (i) activity locations or places [1,2,10], (ii) activity types [36], and (iii) transportation modes [37]. The hereby proposed algorithm aims to contribute to the field of activity place detection and should allow improvements for exposure assessment in health and place studies.

## Methods

### Place detection algorithms

Instead of grouping temporally contiguous and spatially near-by points on a point-by-point basis, the proposed algorithm operates globally by calculating a kernel density surface. This allows deriving a smoothed surface corresponding to the probability density function of a random sample of 2D points, the strength of the smoothing being controlled by the bandwidth. Local density maxima, or peaks, are then retained as candidates for actual stops. GPS points are further allocated either to a peak or to a trip segment. This makes it possible to establish a history of stops and trips. Details of this kernel-density algorithm (A_kd_) and of the classical fixed threshold algorithm (A_ft_) – as presented in [1] – are provided in Additional file 1: Appendix 1. The A_kd_ code is further available as an ArcToolBox for ArcGIS 10 on the Spatial Health Research Lab website (http://www.spherelab.org).

### Track processing

Performance of both algorithms was evaluated using a set of 750 artificial GPS tracks, with three stops per track. Details on artificial track generation are provided in Additional file 1: Appendix 2. Artificial tracks were used because their characteristics, particularly in terms of noise level and stop location and duration, could be controlled. This allowed to precisely evaluate algorithms’ capacity to detect stops, in terms of spatial accuracy – stop location – and temporal accuracy – stop duration. Performance was further evaluated according to GPS track characteristics – noise levels and stop durations – and according to bandwidth value. Both algorithms require definition of a minimal stop duration, which was in our case set to 5 minutes, consequently disregarding shorter stops. The proposed A_kd_ algorithm requires the definition of a kernel bandwidth and the traditional A_ft_ requires the definition of a distance bandwidth. To test parameter sensitivity to parameter value, six bandwidth values were defined as follows: [10; 50; 100; 200; 500; 1000 m]. Choosing the best parameter value may require some trial and error for each new set of data [11]. An algorithm with a low level of sensitivity to parameter value, or, in other words, providing suitable results for a broad range of parameter values, is desirable. All 750 tracks were processed using all six bandwidth values, resulting in the processing of 750*6=4,500 tracks with each algorithm, representing an attempt to detect a total of 4500*2*3=27,000 stops.

### Performance indicators

*Global performance* was measured by computing the number of stops detected per track. Processed tracks would be classified as ‘on target’ when three stops were detected, and ‘false negatives’ or ‘false positives’ when detecting respectively fewer and more than three stops. Tracks with detections of three stops resulting from a combination of false negatives and false positives, i.e. for which distance between a detected stop and the closest true stop was greater than 1,000 m, were considered as outliers and discarded (four cases).

*Spatial accuracy* was established as the Euclidian distance between a detected stop and the closest true stop. This metric was computed for the subset of tracks for which three stops were detected only (the ‘on target’ group).

*Temporal accuracy* was defined as the percentage of over- or underestimation of true duration using (*Δ*_*found*_ − *Δ*_*true*_)/*Δ*_*true*_ where Δ denotes the duration, the reference stop being the closest true stop.

Performance indicators were compiled in relation to GPS noise range – [0; 50], [50, 100], [100; 150] and [150, 200] (bounds in meters) – stop durations – [00:00; 00:20]. [00:20; 02:00] and [02:00; ∞] (bounds expressed in hours and minutes, results not shown for sake of brevity) (see track generation below), and bandwidth – [10;50;100;200;500;1000].

*Capacity to discriminate between two true close stops:* Because of the nature of the A_kd_ algorithm – smoothing the point distribution and possibly joining nearby clusters of points – discriminating close but distinct stops may be a challenge. This may be an even greater challenge with larger kernel bandwidth, i.e. stronger smoothing. We therefore compiled the number of stops correctly identified among tracks that contained two or more neighbouring stops within 800 m, or half a mile.

*Sensitivity to bandwidth choice*: for each of the 750 tracks, the smallest and largest bandwidths for which the right number of stops was detected were recorded and averaged, per noise category. This provided a lower and upper limit of bandwidths for which the right number of stops was predicted. The wider the range, the less the performance was sensitive to bandwidth choice. Because a high sensitivity to parameter choice would require extensive testing and adjustment for new datasets, algorithms with low sensitivity are valuable.

## Results

### Evaluation of stop detection performance

Global performance statistics are shown in Table 1. A_kd_ correctly detected three stops for more than 90% of tracks with bandwidths of 200 and 500 m. With A_ft_, the best performance – correctly detecting three stops for 65.5% of tracks – was obtained with a 1,000 m bandwidth. Yet, A_ft_ generated a high proportion of false negatives and false positives for all bandwidths.

**Table 1:** Global performance– proportion of ‘on target’ tracks or with false negatives or false positives in relation to bandwidth

**Bandwidth**	**Algorithm**					
	***A***_**ft**_			**A**_**kd**_		
	**False neg.**	**On target**	**False pos.**	**False neg.**	**On target**	**False pos.**
10	100.0			86.0	9.9	4.1
50	90.8	3.1	6.1	45.6	42.1	12.3
100	78.9	9.7	11.3	22.9	73.3	3.7
200	54.2	22.8	23.0	7.7	92.3	
500	14.8	54.8	30.4	8.0	92.0	
1 000	11.7	65.5	22.8	12.1	85.6	2.3

### Effect of GPS noise on performance

Logically, highest performance was reached with lower GPS noise levels (0–50) (See Table 2).

**Table 2:** – Performance according to noise at stop – percentage of trips with the right number of stops detected in relation to bandwidth

**Bandwidth**	**Algorithm**							
	***A***_**ft**_				***A***_**kd**_			
	[0. 50]*	**[50. 100]**	**[100. 150]**	**[150. 200]**	**[0. 50]**	**[50. 100]**	**[100. 150]**	**[150. 200]**
10					36.3	6.5		
50	13.7				92.3	59.5	17.4	4.7
100	42.3	1.0			93.5	92.0	65.8	43.8
200	74.9	18.5	4.7		92.9	95.5	92.6	88.0
500	75.0	66.5	51.6	28.1	91.1	93.0	93.2	90.6
1 000	75.6	69.5	59.5	58.3	86.3	85.5	88.4	82.3

A_ft_ did not detect any stops when the bandwidth was smaller than noise. Best performance (75.6% of correctly detected stops) was attained with the largest bandwidth tested (1,000m) and lowest noise range (from 0 to 50m). Decreasing bandwidth or increasing noise resulted in lower performance. For example, performance degraded steeply when using the 200m bandwidth, from 74.9% at a [100, 150] noise range to a mere 4.7% with a [100, 150] noise range.

Meanwhile, A_kd_ attained high ratios of tracks with correct detection of three stops (i.e. around or above 90%) as long as noise was smaller than the kernel bandwidth. Even when noise magnitude was several-fold wider than kernel bandwidth, A_kd_ still succeeded in a few cases to find the right number of stops. It is worth keeping in mind that for this metric, only the number, not the spatial accuracy, of the detected stops was considered.

### Spatial accuracy and duration estimation

**Figure 2:**
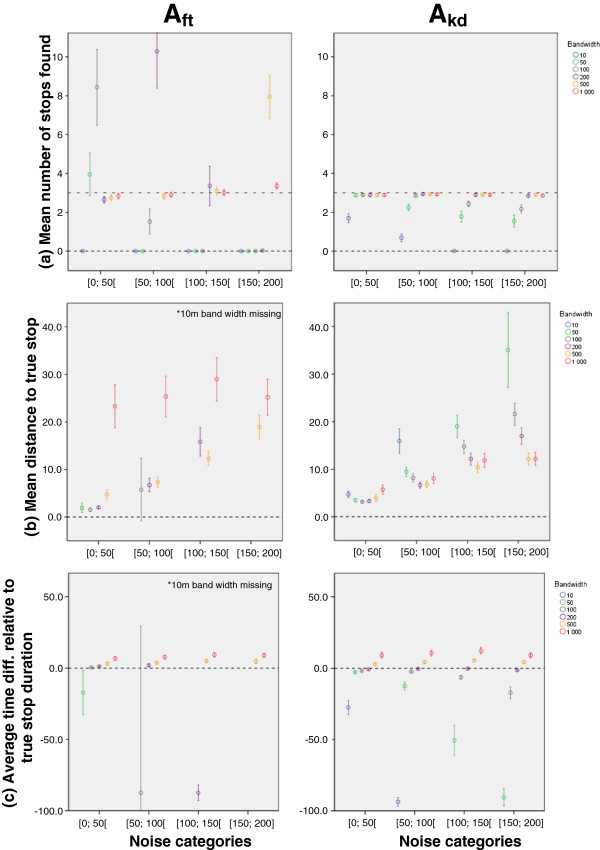
Performance metrics for both algorithms.

Figure 2 shows three graphs for each tested algorithm, illustrating the average number of stops detected per track, the average Euclidian distance between a detected and the closest actual stop, and the average percentage of over/underestimation of stop duration. Performance measures are presented in relation to GPS noise levels (x-axis) and bandwidth (colour symbol).

### Average number of detected stops

A_kd_ slightly underestimated the number of stops, while A_ft_ had a tendency to overestimate them, a known limitation of this class of algorithms [10]. Moreover, A_ft_ presented a much larger variance in performance than A_kd_. For example, with a 200m bandwidth, the average number of detected stops increased from almost 3 (noise<50m) to more than 10 (50m<noise<100m) then back to 3 (100m<noise<150m) but with a much larger variance and finally to no stops detected at all for the highest noise level (150m<noise<200m). A_kd_ exhibited more resilience to noise with a clearer pattern and less degradation of performance with increasing noise.

Graphs b and c in Figure 2 provide statistics for tracks and noise/bandwidth combinations for which three stops were detected, because in the case of a non one-to-one match, spatial correspondence between detected and true stop could have been spurious and could bias results.

### Spatial accuracy

Spatial accuracy was inversely related to GPS noise level. For A_ft_, bandwidth value strongly influenced spatial accuracy. The worst performance was obtained with a 1,000m bandwidth, notwithstanding the noise level. Yet, this same 1,000m bandwidth performed best in terms of number of stops detected. This means that with A_ft_, bandwidth choice represents a trade-off between detecting the right number of stops and positional accuracy.

A_kd_ exhibited a clear homothetic pattern as spatial accuracy degraded much quicker as noise increased when using smaller kernel bandwidths than larger ones. With increasing noise, the average distance between a true and a detected stop rose from around 3.5m to 35.1m using the 50m kernel bandwidth while it only doubled from 5.7m to 12.2m with the 1,000m bandwidth. Whereas A_ft_ yielded the shortest distances between true and detected stops at very low levels of noise (and excluding the 1,000m bandwidth), A_kd_ maintained a better performance across the board with a spatial accuracy below 15m for most bandwidths larger than 50m.

### Temporal accuracy

Overall, larger bandwidths translated in larger overestimation of stop durations. The 200 and 500m parameter values provided the best stop duration estimation and were relatively independent from noise, especially for A_kd_. Smaller bandwidths provided underestimation of stop durations that were larger than the overestimation obtained with larger bandwidths.

### Capacity to discriminate close stops

Table 3 presents the number of tracks for which three stops were correctly detected, among the 88 tracks that had two or more stops within 800m.

**Table 3:** Number of tracks for which three stops were correctly detected among the 88 tracks with two or more stops within 800m

	**Number of correctly identified tracks (three stops)**	
**Bandwidth**	**A**_ft_	**A**_kd_
10	0	8
50	2	32
100	9	54
200	18	60
500	49	35
1000	54	5
Total	132	194

For A_ft_, capacity to discriminate close neighbours increased with growing bandwidths, from no track being correctly classified using a 10m bandwidth to 54 tracks correctly classified (61%) using a 1,000 m bandwidth. For A_kd_, the relationship between bandwidth and discrimination capacity was inverse U-shaped, with a best performance of 60 correctly classified tracks (68%) using a 200m kernel bandwidth.

### Sensitivity to bandwidth choice

**Figure 3:**
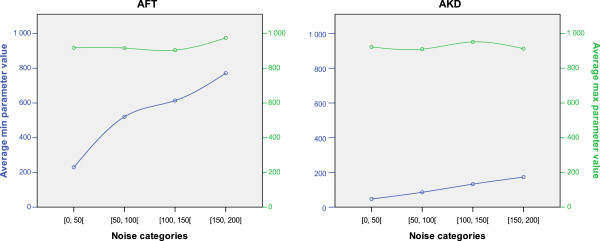
Parameter sensitivity to noise.

For both algorithms, the average of the largest bandwidth values for which three stops were correctly identified exhibited a similar pattern, with a more or less constant average, slightly below 1,000m (See Figure 3). This indicates that noise had a limited impact on the 1,000m bandwidth’s capacity to detect stops, for both algorithms (but, as mentioned above, positional accuracy was affected at larger bandwidths).

Yet, the average of the smallest parameter values for which three stops were correctly identified exhibited contrasting patterns between methods. For A_ft_, the average minimum bandwidth rose steeply with noise, from 229m to 520m, 614m and 771m for each increasing noise category. For A_kd_, values remained much lower, from 47m to 173m, each average minimum value being just below the upper bound of the noise category. The average minimum value was always 4.6 to 6 times larger for A_ft_ than for A_kd_. Consequently, the range of bandwidths to choose from to correctly identify stops was much larger for A_kd_ than for A_ft_. In the highest category of noise, correct performance of A_ft_ was restricted to bandwidths comprised between 771m and 975m whereas A_kd_ would perform well with values between 173m and 912m, providing 3.6 times more headroom. A_kd_ is less sensitive to parameter choice and more resilient to noise.

Some summary findings are provided in Table 4. A_kd_ outperforms A_ft_ for each of the six presented performance measures.

**Table 4:** Summary of performance

**Criteria**	**A**_**ft**_	**Comment**	**A**_**kd**_	**Comment**
Highest proportion of tracks with correctly identified number of stops. depending on parameter value	65.5%	Obtained with 1000 m radius	92.3%	Obtained with 200 m bandwidth
Number of noise/parameter combinations for which detection correctly identifies three stops for at least 70% of tracks (out of 24 combinations)	3	Performance sharply decreasing with increasing noise; best combination yields 75.6% of correct identification of three-stop tracks	15	10 out of these 15 successfull combinations with correct detection of 90% or more of three-stop tracks
Number of correctly identified stops among tracks with close (<800 m) neighbours	132	Larger radii=better prediction	194	Inversed U-shaped relation to bandwidth: best capacity with ‘average’ bandwidth of 200 m
Number of noise/parameter combinations for which the average number of detected stops is around 3 (2.8<average<3.2)	6	10 noise/parameter combinations for which average=zero	15	2 noise/parameter combinations for which average=zero
Number of noise/parameter combinations for which distance between detected and true stop is less than 15 m in average (out of 24 combinations)	8	Standard-errors larger in AFT than in AKD for all combinations	17	11 combinations with less than 10 m in average
Number of noise/parameter combinations with duration difference between detected and true stop less than 10% error	11	AKD outperforms AFT for 16 out of 24 combinations	16	Duration difference below 5% for 200 m bandwidth at all noise levels

Akd/Aft comparison.

## Discussion

A new algorithm to extract significant places and derive a timetable of visits from raw GPS data based on kernel density computation is assessed in comparison to a more ‘classical’ distance threshold algorithm. Artificial tracks with known characteristics are processed and allow precise performance evaluation. Motivations driving the development of this algorithm were both practical – that is, to provide an efficient solution to derive activity locations from GPS datasets in order to improve the characterization of activity spaces and related environmental exposures in epidemiological modelling – and technical, by trying to offer a solution for which the sensitivity of the algorithm parameter choice was low, i.e. where a broad range of parameter values would perform well under various conditions. An algorithm whose performance is less sensitive to parameter choice or noise is also important because noise can vary depending on built environment characteristics. Longer stays in high-rise central areas or urban canyons generally generate subsets of data points with large noise. Other methods such as learning algorithms [12], which were not discussed here, may provide interesting results without fiddling with parameter adjustments. Yet, such algorithms require to be trained beforehand on sample data and their setup is generally more complicated. The proposed algorithm offers a good balance between the simplicity of use of the fixed threshold approach and the performance of more advanced and more technical solutions, such as learning algorithms.

Experiments on randomly generated synthetic GPS tracks showed the proposed algorithm outperformed the fixed threshold algorithm for almost all indicators, correctly identifying the three artificially generated stops with varying duration and noise levels more than twice as often (2,964 cases against 1,169, on a total of 4,500). Similarly, although A_ft_ had the best spatial accuracy with smaller bandwidths and for the lowest noise levels, A_kd_ succeeded in maintaining a better overall accuracy across all bandwidths and noise categories. Stop duration estimation was very accurate, although smaller bandwidths systematically provided underestimation.

We believe the methods presented here are useful for three reasons. First, the proposed method for artificial track generation allows control over various parameters such as GPS noise at stop locations and stop durations. This makes it possible to precisely evaluate the performance of a given algorithm in relation to these characteristics. We welcome application of this methodology which would optimize comparison with other algorithms, and allows testing of performance under a wide range of controlled conditions. The Python code for automatic generation of artificial tracks as presented here is made available on the authors’ lab website at http://www.spherelab.org/tools. Second, the proposed set of performance indicators is useful for algorithm evaluation. Looking at one performance criteria only (such as the number of detected stops for example) may be misleading, as trade-off sometimes occurs, for example between spatial and temporal accuracy. Third, the proposed kernel-based algorithm has outperformed the more ‘traditional’ fixed-threshold method along all measured performance indicators. Because of its ease of implementation, we recommend its use for activity location detection in health research. To facilitate usage, an ArcToolbox version of the algorithm for ArcGIS 10 is made available on the authors’ website. We welcome proposals for improvement of this algorithm and will maintain history of versions on our website.

### Limitations

Whereas the use of artificial tracks offers advantages, some limitations need to be acknowledged. Because place extraction is based on point density, such algorithms will not perform as well if points are not sampled continuously. In real-world situations, GPS signals can be interrupted, particularly in urban areas where people spend time inside buildings that hinder signal reception. This shortcoming requires filling data gaps using techniques such as interpolation. This was not an issue in the context of this paper since the synthetic tracks did not contain signal drops. In our own experiments conducted with real-life GPS data, a simple linear interpolation was used and gave satisfactory results. More sophisticated approaches, such as interpolation along a network, could prove useful, and decision rules to optimize interpolation, for example using time and distance thresholds between two collected consecutive GPS data points, need to be evaluated.

Another limitation in the work presented here is the use of a constant travelling speed (roughly 36 km/h) for the trip sections of the tracks; no speed variation was introduced to simulate change of transportation mode or traffic slow-downs for example. This was done to limit the number of varying factors. However, slower speeds should not influence significantly the stop detection capacity. Indeed, empirical tests on real-life GPS tracks showed that the main issue for automatic stop detection was related to signal noise measured at the stop itself.

Another limitation of artificial tracks is the choice of a normally distributed random noise, which may be viewed as an oversimplification of true GPS noise, particularly in an urban context where bad satellite reception can lead to systematic errors in the calculated position. However, actual GPS fixes provide quality information along latitude and longitude (e.g. Dilution Of Precision values, number of satellites used) that can help filter out suspicious fixes.

Finally, one shortcoming of the proposed algorithm is that because GPS points are processed globally, real-time processing may be less efficient, although periodical re-running of the algorithm or treatment of data subsets may prove efficient.

## Conclusions

The proposed novel kernel-based algorithm performed better than its classic counterpart on a set of synthetic tracks with varying stop durations and noise levels. Yet, further validation with real-world tracks, covering a variety of contexts, both in terms of urban environments and mobility patterns, are required. However, in order to be able to be considered as a ‘gold standard’ source to document stop locations, collected GPS tracks should actually be post-processed for validation of stops – and trips and transportation modes – by people who wore the GPS units. Prompted recall applications allowing participants to visualize their tracks and confirm/infirm stop locations and transportation modes are required. Only artificial tracks with known characteristics or real-life tracks with post validation through prompted recall surveys are useful to truly test the performance of such algorithms. In short, true stops need to be known to allow identification of true/false positives or negatives following track processing.

Increasing availability and use of GPS devices opens great opportunities for mobile health applications. Yet, well-performing and validated algorithms are required to correctly identify activity locations, trips and transportation modes from raw GPS datasets, both for health research and for other fields increasingly considering mobility patterns. We therefore recommend further developments in the area of activity place, travel mode and activity type detection. Such efforts require the constitution of comprehensive datasets including both raw GPS tracks and prompted recall validations of itineraries and activities.

## Competing interests

The authors declare that they have no competing interests.

## Authors’ contributions

YK and BT designed the algorithm and study. BT ran the analyses and drafted the manuscript. All authors critically revised the manuscript and read and approved the final version.

## Supplementary Material

Additional file 1: Appendix 1 Description of the two tested algorithms. **Appendix 2.** Artificial GPS track generation. Click here for file
