# Supplementary material for: Detecting activity locations from raw GPS data: a novel kernel-based algorithm
Source: Int J Health Geogr. 2013 Mar 16;12:14. doi: 10.1186/1476-072X-12-14 (PMC3637118; doi:10.1186/1476-072X-12-14)
Supplement: Additional file 1: Appendix 1 — Description of the two tested algorithms. Appendix 2. Artificial GPS track generation. [file 1476-072X-12-14-S1.docx]

# Additional online material

# Appendix 1: Description of the two tested algorithms

# Appendix 2: Artificial GPS track generation

# Appendix 1: Description of the two tested algorithms.

## Details of Hariharan activity place extraction algorithm

Step-by-step description of the fixed threshold algorithm, as presented in Hariharan et al. [[1](#_ENREF_1)], noted *Aft*:

1. Define *Diameter* function to compute the greatest distance between all pairs of points in a set
2. Define *Medoid* function to compute the centroid of a point dataset
3. For each point P_i_ in the GPS point dataset, do:
   1. Find the next point P_j_ so that P_j_ – P_i_ ≥ ∆_duration_
   2. Check if *Diameter*(P_i_, P_j_) > ∆_roam_, in which case continue to point P_i+1_
   3. Otherwise, do:
      1. Select all following points P_j_’ so that *Diameter*(P_i_, P_j_’) ≤ ∆_roam_
      2. Extract stop S = *Medoid*(P_i_, P_j_’), with start and stop times t_i_ and t_j_’
      3. Continue to point P_j+1_’

## Details of the SPHERELab GPS activity place extraction algorithm

Step-by-step description of the kernel-density algorithm, noted *A*_kd_:

1. KD = kernelDensity(Ψ, β): A kernel density surface was calculated from the GPS point dataset Ψ using a quadratic kernel function with a fixed bandwidth *β* using ArcGIS kernel density tool. The resulting raster layer had a 10m square cell size and all cells with null-value were excluded from further analyses 🡪 **KD**.
2. HS = localMaxima(KD): Kernel density raster cells with a value lower than the global mean of the kernel density raster were excluded 🡪 **KD*_f_***. Watershed analyses (using ArcGIS Spatial Analyst) were run on the inversed surface to determine flow directions and sinks. Sinks corresponded to local density maxima. Raster sinks were then converted to vector points, which correspond to the detected activity places 🡪 **HS**.
3. ZHS = findZoneHS(KD*_f_*): From the filtered kernel density raster, we delineated the zone around each visited place within which GPS points contributed to the local density maximum, using the ArcGIS Basin Tool 🡪 **ZHS**.
4. Ψ_1_ = findAllocation_1stPass(Ψ, KD*_f_*, ZHS, γ_1_): Zonal mean *µ_z_* and standard deviation *σ_z_* of kernel densities were calculated within each zone separately. These values were used to normalize the kernel density of each cell so that *nK* = (*K* – *µ_z_*) / *σ_z_* 🡪 **nKD*_z_***. Normalized kernel density, as well as zone identifier from **ZHS** raster, was then extracted to GPS point dataset Ψ through a spatial overlay. A convolution filter γ_1_ was then applied to the temporal sequence of GPS points, for smoothing of the normalized kernel density of each GPS point so that the normalized kernel value of past and following GPS points are taken in account, akin to a moving average of kernel density values. Filter γ_1_ was defined with the following coefficients: [0.1; 0.25; 0.3; 0.25; 0.1]. At this stage, each GPS point carried a tuple consisting of three values (*nK*, *nK_c_*, *ZHS*) corresponding respectively to the normalized kernel density, the convolved normalized kernel density and the zone identifier. Allocation of a GPS point *ψ* to a visited place was then based on the following rule:

if *nK* ≥ 1 OR *nK_c_* ≥ 1 then allocate *ψ* to corresponding visited place [R_1_].

Allocated points were marked with flag *f_alloc_* = 1, while unallocated points were set to a null value.

1. Ψ_2_ = findAllocation_2ndPass(Ψ_1_, γ_2_): A second pass to further refine allocation of GPS points Ψ_1_ was done to catch unallocated points with allocated neighbours, which may typically happen for GPS spike errors. Convolution filter γ_2_, with coefficients [0.5; 1; 1.5; 1; 0.5] was applied to the allocation flag *f_alloc_*. Allocation of a GPS point *ψ* to a visited place ws then decided based on the second rule:

if *f_alloc_* > 0 then allocate *ψ* to corresponding visited place [R_2_].

1. THS = findDuration(Ψ_2_): Once the set of allocated GPS points Ψ_2_ was established, the time spent at each visited place was computed by iterating over Ψ_2_ to find the starting and ending points of all contiguously allocated subsets of GPS points. Stop durations were derived from the timestamps for the corresponding visited place 🡪 **THS**.

NB: The kernel density algorithm presented here is offered online as an ArcToolbox for ArcGIS 10 on the authors’ website. Please go to [www.spherelab.org](http://www.spherelab.org), tools section.

# Appendix 2: artificial GPS track generation

The procedure to build the sets was adapted from Kami et al. [[2](#_ENREF_2)]:

1. Random selection of 750 pairs of coordinates within the Montreal Metropolitan Area; 2. Calculation of shortest road network for each pair of coordinates using Network Analyst extension in ArcGIS 10;

3. Transformation of resulting polylines into points, with a regular spacing of 10 m – corresponding to a travel speed of 36 km/h using a tracking device set at a one second epoch;

4. Random shift of points along X and Y axes to mimic imprecision of GPS tracks along followed routes; the shift following a bi-dimensional normal distribution centered on 0 with a standard deviation of 1.25 m;

5. Random selection of three data points per track to serve as stop locations;

6. Determination of stop durations λ_d_ using a Poisson distribution, with a seed value for the three stops randomly chosen from one of the three following normal distributions (mean ± stdev): (i) 10min ± 3min, (ii) 1h ± 15min and (iii) 5h ± 1h – these three classes of duration respectively representing typical short- (e.g. dropping kids at the kindergarten), medium- (e.g. going to the swimming pool) and long-term (e.g. work hours) activities.

7. Generation of additional stop points (1 point per second) with simulated noise along X and Y axes - noise added by shifting coordinates from original stop point using a normal distribution with mean 0 and standard deviation σ_n_, σ_n_ being randomly drawn from a uniform distribution ranging from 10 to 200 m. Performance indicators are presented by groups of noise range – [0; 50[, [50, 100[, [100; 150[ and [150, 200] – bounds in meters.

Varying the two parameters λ_d_ and σ_n_ allowed to test the performance of algorithms in relation to stop duration and levels of GPS noise during stop periods.

NB: The algorithm for artificial track generation is available online as a Python code on the authors’ website. Please go to [www.spherelab.org](http://www.spherelab.org), tools section.

1. Hariharan R, Toyama K: **Project Lachesis: parsing and modeling location histories**. *Geographic Information Science* 2004:106-124.

2. Kami N, Enomoto N, Baba T, Yoshikawa T: **Algorithm for detecting significant locations from raw GPS data**. In: *Discovery Science: 2010*: Springer; 2010: 221-235.
